# The effects of physical activity interventions on self-esteem during and after cancer treatment: a systematic review and meta-analysis

**DOI:** 10.1038/s41598-024-74888-2

**Published:** 2024-11-06

**Authors:** Andrea Rodriguez-Solana, Luis Gracia-Marco, Cristina Cadenas-Sanchez, Andrés Redondo-Tébar, Andres Marmol-Perez, Jose Juan Gil-Cosano, Francisco J. Llorente-Cantarero, Esther Ubago-Guisado

**Affiliations:** 1 Department of Physical Education and Sports, Faculty of Sport Sciences, Sport, and Health University Research Institute (iMUDS), University of Granada, Granada, 18007 Spain; 2 Instituto de Investigación Biosanitaria, ibs.Granada, Granada, 18012 Spain; 3 Centro de Investigación Biomédica en Red Fisiopatología de la Obesidad y Nutrición (CIBERobn), Instituto de Salud Carlos III, Madrid, 28029 Spain; 4 Social and Health Research Center, Universidad de Castilla-La Mancha, Cuenca, 13071, Spain; 5 Department of Epidemiology and Cancer Control, St. Jude Children’s Research Hospital, Memphis, TN USA; 6 Department of Health Sciences and Biomedicine, Faculty of Health Sciences, Universidad Loyola Andalucía, Sevilla, 41703 Spain; 7 Instituto de Investigación Biomédica Maimonides (IMIBIC), Córdoba, 14004 Spain; 8 Departamento de Didácticas Específicas, Facultad de Educación, Universidad de Córdoba, Córdoba, 14071 Spain

**Keywords:** Exercise interventions, Mental health, Cancer, Children, Adolescents, Adults, Cancer, Psychology, Diseases, Oncology

## Abstract

To investigate the effect of physical activity (PA) (both general and its type) on self-esteem during and after cancer treatment. A systematic search was conducted across PubMed, Web of Science, Scopus, SPORTDiscuss, and PsycINFO from their inception to February 2024. The systematic review included 32 studies, with 15 studies (13 RCT and 2 quasi-experimental) and 3604 participants (66.7% female) included in the meta-analysis involving controlled trials using a control group and at least one PA intervention group. The study was registered in PROSPERO (CRD42022309771). Risk of bias for RCTs was assessed using the Cochrane Collaboration’s tool for assessing risk of bias (RoB2), and quasi-experimental studies with the Joanna Briggs Institute critical appraisal tool. PA significantly improved self-esteem during and after cancer treatment (pooled SMD = 0.32, p < 0.01). Specifically, aerobic PA (pooled SMD = 0.33, p = 0.04) and mind-body exercise (pooled SMD = 0.70, p = 0.03) had positive effects on self-esteem. Overall, PA interventions improved self-esteem during cancer treatment (pooled SMD = 0.50, p = 0.01) and in PA interventions lasting more than 12 weeks (pooled SMD = 0.44, p = 0.02). In conclusion, PA (specifically, aerobic and mind-body exercises) may have a positive effect on self-esteem during and after cancer treatment, with cancer status and the duration of the intervention being key factors.

**Supplementary Information:**

The online version contains supplementary material available at 10.1038/s41598-024-74888-2.

## Background

Cancer is slightly more common in men than women (40,9% vs. 39.1%) and remains one of the leading global causes of mortality^1^. The five-year relative survival rate is approximately 68%^1^ but surviving cancer and undergoing cancer-related treatment increases the risk of side effects, such as impaired growth in paediatric population, cardiovascular disease, and secondary malignancy^2–4^.

Individuals during and after cancer treatment may experience psychological issues that contribute to maladaptive lifestyle habits, such as sedentarism and alcoholism^5^, as well as impaired social functioning (e.g., difficulties in school or employment), anxiety, depression, and fear of recurrence^4,6,7^. These psychological sequels, affecting emotional well-being, can lead to changes in self-esteem levels^6^. A study of young adults after cancer treatment^8^ found that low self-esteem, defined as a score of ≤ 25 score on The Rosenberg Self-Esteem scale, was present in 10% of the participants.

Self-esteem is one component of self-perception, alongside self-concept. While self-concept refers to how we describe ourselves, self-esteem relates to how we assess that self-concept, either positively or negatively^9,10^. High self-esteem is associated with better physical and psychological health, academic performance, and quality of interpersonal relationships^9,11^. In contrast, low self-esteem is linked to dissatisfaction, self-loathing, self-contempt, and self-rejection^9^. Factors that can influence self-esteem include negative body image and personal experiences. Self-esteem develops gradually over time, shaped by social interactions and life experiences^12^. It tends to be high during childhood, declines until adolescence^13,14^, rises from mid-adolescence to mid-adulthood, peaks between the ages of 50 and 60, and eventually declines in older age^15^.

The benefits of physical activity (PA) in healthy population are well established^16^. After cancer treatment, PA may not only improve fitness and quality of life but may also reduce depression, psychosocial distress, and recurrence of cancer^17^. Previous research has shown that physical exercise may be safe during and after cancer treatments^18^. However, a more recent study highlights that there is insufficient research on the potential harms of PA to make fully evidence-based risk-benefit assessments for its prescription during cancer treatment^19,20^. Previous studies have shown that different types of PA can reduce depression, anxiety, and fatigue during and after cancer treatment^21,22^. Additionally, while some research found associations between PA interventions and improved self-esteem during and after cancer treatment^23–25^, this area has been less extensively explored. A comprehensive compilation of available studies through a systematic review and meta-analysis is needed. Thus, to the best of our knowledge, this is the first systematic review and meta-analysis aimed at examining the effects of PA interventions (both general and by type) on self-esteem during and after cancer treatment.

## Methods

### Protocol and registration

This study was conducted following the Preferred Reporting Items for Systematic Reviews and Meta-Analysis PRISMA guidelines and PRISMA-S^26,27^ (Supplementary table S1 and Supplementary Table S2). The systematic review and meta-analysis were registered in the International Prospective Register of Systematic Reviews in 2022, with an update made in 2024 (registration number: CRD42022309771). The update was performed through email alerts and by reapplying the search strategy over the past two years to identify any newly published articles.

### Data sources

A systematic search was conducted using MEDLINE (via PubMed), Web of Science (Clarivate), Scopus (Elsevier), SPORTDiscuss (EBSCOhost) and Psycinfo (Ovid) from database inception to February 2024. The search strategy used for each database and the search terms used are available in Supplementary Material (Table S3) which was carried out in parallel with a previous study and was adapted to the subject matter of this study.

### Eligibility criteria

Two reviewers (A.R-S and A.R-T) independently screened and identified studies that potentially met the inclusion criteria. Any disagreements were resolved through consensus, or if necessary, with the involvement of a third researcher (E.U-G). The inclusion criteria were defined as follows: (a) Population: individuals during and after cancer treatment; (b) Age: all age groups; (c) Cancer types: all types of cancers; (d) Study design: observational and experimental studies; (e) Outcome: self-esteem measured using any validated questionnaire; (f) Intervention: any form of PA; (g) Control: groups without a PA intervention (including flexibility-focused activities); (h) Language: studies written in English or Spanish. Exclusion criteria included non-eligible publication types, such as conference proceedings, theses, editorials, letters to the editor, systematic reviews, and meta-analyses.

### Study selection

**Figure 1:**
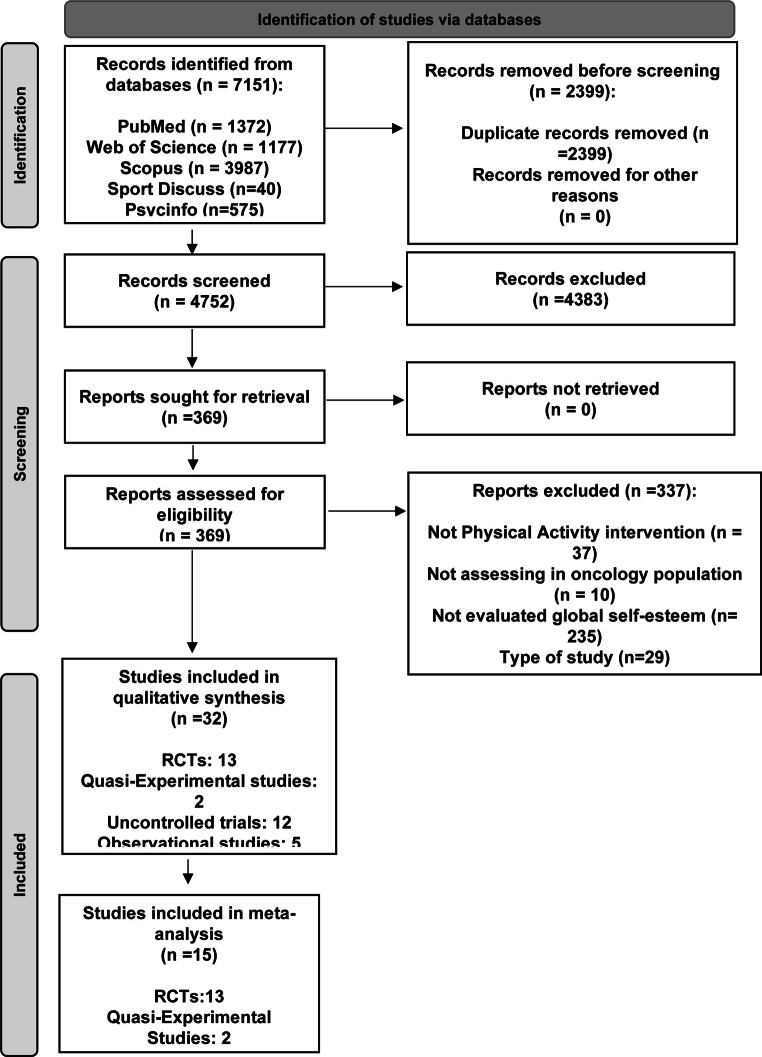
Preferred Reporting Items for Systematic Reviews and Meta-Analyses (PRISMA) flow diagram of study selection.

The study selection process was carried out in several steps. First, records were identified through database searches and duplicates were removed using Endnote X7 0.1. Secondly, titles and abstracts were screened to determine their potential eligibility. Articles that appeared eligible were then read in full to decide on their final inclusion or exclusion in the systematic review and meta-analysis. All steps were completed and reviewed by two investigators (A.R-S and A.R-T). Disagreements were resolved through discussion, adhering to the established inclusion and exclusion criteria. When the inclusion status of a study was unclear, a third reviewer (E.U-G) was involved to reach through discussion. Figure 1 presents the Preferred Reporting Items for Systematic Reviews and Meta-Analyses (PRISMA) flow diagram of the study selection process. Finally, reference lists of the included articles were examined for other relevant studies. Authors of articles with missing data were contacted, and 2 of the 7 studies that had not reported the required information responded and provided the necessary data. Additionally, efforts were made to obtain the full text of certain articles by contacting the respective authors (27 in total); however, the majority (21 authors) did not respond to our requests. A citation index and email alerts were established to track potential new studies published during the course of this study.

###  Classification as ‘during’ or ‘after’ cancer treatment

Studies involving patients receiving any form of cancer treatment, whether as initial cancer therapy or for metastasis or cancer recurrence, were classified as ‘during’ treatment. Studies that included patients not currently undergoing any cancer treatment or receiving androgen suppression therapy or hormone therapy without any other cancer treatment, were defined as ‘after’ treatment. Studies including both types of patients were categorized as ‘both’.

### Risk-of bias assessment

The Cochrane Collaboration’s tool for assessing risk of bias (RoB2) was used for randomized controlled trials (RCTs)^28^. This tool evaluated five domains: randomization process, deviations from intended interventions, missing outcome data, measurement of the outcome, and selection of the reported result. Overall, a study is considered to have a “low risk of bias” if all domains are rated as “low risk”, “some concerns” if at least one domain is rated as “some concern”, and “high risk of bias” if at least one domain is rated as “high risk”, or if multiple domains are rated as “some concerns”.

The Joanna Briggs Institute critical appraisal tool was used to evaluated the quality of quasi-experimental Studies^29^. This tool assesses nine domains: the cause and effect of variables, similar comparison groups and treatment/care, control group, multiple measurements of outcomes, follow-up, similar measurements of outcomes in the different group, outcome measurements in a reliable way, and statistical analysis. Each domain is rated with one of four responses: “yes” (criterion met), “no” (criterion not met), “unclear”, or “not applicable” (N/A). A study was classified as “high quality” if it achieved a quality score of at least 0.75 (i.e., 75%), and as “low quality” if the score was below 0.75. Additionally, a score for each criterion was calculated by dividing the number of positive ratings by the total number of studies evaluated, providing an overview of how well the current literature performs on each criterion. Two researchers (A.R-S and A.R-T) independently assessed the risk of bias to determine the quality of the included studies, with any discrepancies resolved by a third reviewer (E.U-G).

### Data extraction

Articles retrieved from the databases were exported and managed using an EndNote library (Endnote version X7.0.1). Data extracted from the original reports, based on the inclusion and exclusion criteria, included: (a) first author and year of publication; (b) country of data collection; (c) study design; (d) sample characteristics; (e) method used for measuring self-esteem at baseline and follow-up; (f) type of control group intervention; and (g) type of PA intervention. Data extraction was independently verified by two researchers (A.R-S and A.R-T), and any discrepancies were resolved through consensus with a third researcher (E.U-G).

### Statistical considerations

The DerSimonian and Laird method was used to compute Standardized Mean Difference (SMD) and 95% confidence intervals (95% CIs), as the summary measure. For data synthesis and meta-analysis, random-effects models were employed. When studies provided mean self-esteem values at baseline and endpoint or reported mean value changes, SMD was calculated. SMD of 0.2 to 0.5 were considered small, 0.5 to 0.8 were considered medium, and values greater than 0.8 were considered large^30,31^. The heterogeneity of results across studies was assessed using the I^2^ statistic^32^. In addition, exploratory subgroup analyses were performed to examine how the intervention affects self-esteem depending on the type of PA (aerobic PA, resistance training, combined PA, and mind-body exercise), cancer status (during and after cancer treatments), and lasting of the intervention (12 weeks or less and more than 12 weeks). Furthermore, exploratory subgroup analyses were conducted to explore differences across groups of age (children and adolescents under 18 years of age and adults with 18 years of age or older), study design (randomized controlled trial and quasi-experimental study), and self-esteem questionnaires (Rosenberg self-esteem scale and other than Rosenberg self-esteem scale questionnaires). Funnel plots were examined to assess the risk of potential publication bias, with Egger’s regression asymmetry test used to detect asymmetry. Further, the ‘trim and fill’ procedure^33^ was also applied to identify and correct for funnel plot asymmetry potentially due to publication bias. A leave-one-out cross-validation analysis was performed to evaluate the impact of excluding individual studies on the combined pooled SMD by sequentially omitting one study at a time. The summary measure used in this study was the SMD.

Statistical analyses were performed using Comprehensive Meta-Analysis software version 2.2 (Biostat Inc., Englewood, NJ, USA), with statistical significance set at *p* < 0.05.

### Classification of PA interventions

Due to the diversity of PA interventions, they were classified into four categories: aerobic PA, resistance training, combined PA, and mind-body exercise. Aerobic PA interventions include belly dance, treadmill, elliptical, and walking. Resistance training encompasses exercises like leg extensions, leg curls, leg presses, calf raises, chest presses, seated rowing, triceps extensions, biceps curls, and modified curl-ups. Combined PA includes a variety of sports and recreational activities, as well as programs combining aerobic and resistance training. Mind-body exercise refers to practices such as yoga and Pilates.

## Results

### Study selection and adverse effects

A total of 7151 studies were identified from the literature search, of which 2399 were excluded before screening due to duplication. After screening by title and abstract, 369 full-text articles were reviewed for eligibility. Finally, 32 studies were included in the systematic review, of which 13 RCT’s and 2 Quasi-Experimental Studies were included in the meta-analysis (Fig. 1).

In this systematic review and meta-analysis, 13 studies reported “no significant adverse effects” while 20 studies did not provide information on whether any adverse effects were observed.

### Risk-of bias assessment

The quality of the RCTs included in the meta-analysis (*n* = 13, Table S4) showed that six studies (46.2%) had a low risk of bias, while seven studies (53.8%) had some concerns. In terms of specific domains, all studies were rated as low risk for the randomization process, missing outcome data, and measurement of the outcome (100%). For deviations from intended interventions, eight studies (61.5%) were rated as low risk, and five studies (38.5%) were rated as having some concerns. Regarding the selection of reported results, ten studies (76.9%) were rated as low risk, and three studies (23.1%) had some concerns. Of these 13 articles, 62% were analyzed using intention-to-treat principle, while 38% were analyzed using per-protocol principle.

The risk of bias in the quasi-experimental studies (*n* = 2, Table S4) indicated that both studies had high-quality scores. In terms of specific domains, 100% of the studies met the methodological quality criteria for the cause and effect of variables, similar treatment/care groups, presence of a control group, multiple measurements of outcomes, consistency of outcomes measurements across groups, reliability of outcome measurements, and statistical analysis. For the domain of similar comparison groups, one study did not meet the methodological quality criterion (50%), while the other was rated as unclear (50%). In the follow-up domain, one study was rated as unclear (50%), and the other met the methodological quality criterion (50%). Both of these articles (100%) were analyzed using per-protocol principle.

### Study characteristics

Table 1 present the characteristics of the studies included in the systematic review and meta-analysis. A total of 3604 participants (66.7% female) during or after cancer treatment were involved in the select studies of this systematic review. These studies were conducted in 12 different countries, with participants having the following cancer types: Ewing sarcoma (*n* = 1), Testicular cancer (*n* = 1), Breast cancer (*n* = 17), Rectal cancer (*n* = 1), and various malignancy disease types (*n* = 13). The age of the participants ranges from 8 years and older, with the sample sizes varying between 16 and 618 (median = 107 participants). Regarding self-esteem measurements, 24 studies (72.7%) used the Rosenberg Self-Esteem Scale, three used the Physical Self-Inventory (PSI) (9.1%), three (9.1%) the Physical self-perception Profile, two (6.1%) the KINDL questionnaire, and one (3%) the Self-esteem questionnaire (SEQ-42). Despite the variety of questionnaires, all studies in this meta-analysis provided self-esteem scores.

**Table 1:** Characteristics of studies included in the systematic review and meta-analysis.

Study characteristics		Population characteristics at baseline				Outcome			
**Authors and year**	**Country**	**Age**	**Sample size** **[n (% male)]**	**Cancer-type**	**Time period**	**Method**	**Baseline** **(mean** ± SD)	**Follow-up** **(mean** ± SD)	
*Randomized Controlled Trials [n = 14]*									
Adams et al. 2018^34^	Canada	43.7 ± 10.8	63 (100% male) Walking: 35 CG: 28	Testicular cancer	After cancer treatment	The Rosenberg Self-Esteem Scale	Walking: 32.5 ± 5.5 CG: 36.0 ± 4.8	Walking: 34.5 ± 4.1 CG: 35.0 ± 5.0	
Boing et al. 2023^35^	Brazil	18 years or older	52 (100% female) Pilates: 18 Belly: 18 CG:16	Breast cancer	After cancer treatment	The Rosenberg Self-Esteem Scale	Pilates: 30.2 ± 1.1 Belly dance: 32 ± 1.4 CG: 30.1 ± 1.7	Pilates: 32.6 ± 1.2 Belly dance: 33 ± 0.9 CG: 32 ± 1.1	
Cadmus et al. 2009 ^1^^36^	USA	35–75	50 (100% female) Sports/recreational activities: 25 CG: 25	Breast cancer	During cancer treatment	The Rosenberg Self-Esteem Scale	Sports/recreational activities: 34.8 ± 4.2 CG: 35.2 ± 3.8	Sports/recreational activities: 34.3 ± 4.9 CG: 34.5 ± 3.6	
Cadmus et al. 2009 ^2^^36^	USA	40–75	74 (100% female) Sports/recreational activities: 37 CG: 37	Breast cancer	After cancer treatment	The Rosenberg Self-Esteem Scale	Sports/recreational activities: 34.2 ± 5.5 CG: 33.2 ± 5.7	Sports/recreational activities: 34.5 ± 5.2 CG: 33.4 ± 5.9	
Courneya et al. 2007^37^	Canada	25–78 (Mean 49 years)	242 (100% female) Resistance training: 82 Aerobic physical activity: 78 CG: 82	Breast cancer	During cancer treatment	The Rosenberg Self-Esteem Scale	Resistance training: 34.1 ± 4.2 Aerobic physical activity: 34.0 ± 5.1 CG: 34.1 ± 4.6	Resistance training: 34.7 ± 4.2 Aerobic physical activity: 34.5 ± 5.1 CG: 33.2 ± 5.5	
Fretta et al. 2021^38^	Brazil	55.3 ± 11	34 (100% female) Pilates: 18 CG: 16	Breast cancer	After cancer treatment	The Rosenberg Self-Esteem Scale	Pilates: 30.4 ± 5.1 CG: 30.5 ± 6.6	Pilates: 35.1 ± 3.8 CG: 33.1 ± 4.3	
Gokal et al. 2016^39^	UK	18–75	50 (100% female) Walking: 25 CG: 25	Breast cancer	During cancer treatment	The Rosenberg Self-Esteem Scale	Walking: 21.7 ± 4.4 CG: 20.4 ± 4.9	Walking: 23.8 ± 4.6 CG: 19.5 ± 4.2	
Kovačič et al. 2011^24^	Slovenia	≥ 40	32 (100% female) Yoga: 16 CG: 16	Breast cancer	During cancer treatment	The Rosenberg Self-Esteem Scale	Yoga: 21.3 ± 1.3 CG: 21.2 ± 1.4	Yoga: 23.7 ± 1.1 CG: 21.2 ± 1.7	
Leite et al. 2021^25^	Brazil	55 ± 10	52 (100% female) Belly dance: 18 Mat Pilates: 18 CG: 16	Breast cancer	After cancer treatment	The Rosenberg Self-Esteem Scale	Belly dance: 32.1 ± 1.3 Mat Pilates: 30.4 ± 1.2 CG: 31.9 ± 1.4	Belly dance: 33.3 ± 1.0 Mat Pilates:32.7 ± 1.0 CG: 32.2 ± 1.1	
Musanti 2012^40^	USA	50.5 ± 7.5	55 (100% female) Aerobic physical activity: 12 Resistance training: 17 Combined physical activity: 13 CG*: 13	Breast cancer	After cancer treatment	The Rosenberg Self-Esteem Scale	Aerobic physical activity: 23 ± 6.6 Resistance training: 25.3 ± 3.2 Combined physical activity: 24.4 ± 4.8 CG*: 25.7 ± 4.0	Aerobic physical activity: 21.7 ± 4.4 Resistance training: 26.4 ± 2.6 Combined physical activity: 23.6 ± 1.2 CG*: 26.3 ± 3.9	
Rastogi et al. 2020^41^	USA	54.4 ± 11.2	48 (96% female) Combined physical activity: 26 CG: 22	Breast and colorectal cancer	After cancer treatment	The Rosenberg Self-Esteem Scale	Combined physical activity: 22.6 ± 3.4 CG: 20.2 ± 3.7	Combined physical activity: 23 ± 4.2 CG: 20.2 ± 4.7	
Saultier et al. 2021^23^	France	10.4 ± 0.5	70 (42.5% female) Combined physical activity: 37 CG :33	Various cancer types	During cancer treatment	Self-esteem with the “Physical Self-Inventory—Very Short Form” (PSI-VSF)	Combined physical activity: 4.4 ± 0.2 CG: 4.6 ± 0.1	Combined physical activity: 5.0 ± 0.1 CG: 4.8 ± 0.1	
Van Dijk-Lokkart et al. 2016^42^	Netherlands	8–18	68 (46.7% female) Combined physical activity:30 CG: 38	Various cancer types	During cancer treatment	Global Self-worth with the Dutch versions of the Self Perception Profile for children and adolescents	Combined physical activity: 61.4 ± 29.6 CG: 61.3 ± 28.6	Combined physical activity: 73 ± 25.6 CG: 64.7 ± 33.1	
Wurz et al. 2019^43^	Canada	32.3 ± 7.8	16 (85.7% female) Combined physical activity: 7 GC: 9	Various malignancy disease types	After cancer treatment	The Rosenberg Self-Esteem Scale	Combined physical activity: 29.1 ± 2.2 CG: 28.1 ± 5.5	Combined physical activity: 29.5 29.5 ± 2.3 CG: 28.56 ± 5.8	
*Quasi-Experimental studies [n = 2]*									
Carminatti et al. 2019^44^	Brazil	54.5 ± 8.3	19 (100% female) Belly: 11 CG: 8	Breast cancer	During cancer treatment	The Rosenberg Self-Esteem Scale	Belly: 29 ± 1 CG: 32 ± 1		Belly: 32 ± 2 CG: 32 ± 1
Rosenberg et al. 2014^45^	USA	30.6	199 (82.9% female) Outdoor adventure 1: 87 Outdoor adventure 2: 41 CG: 71	Various malignancy disease types	After cancer treatment	Self-esteem with the Psychological Screening Inventory-2	Outdoor adventure 1: 52.2 ± 10.3 Outdoor adventure 2: 52.3 ± 9.8 CG: 53.8 ± 11.2		Outdoor adventure 1: 50.4 ± 8.9 Outdoor adventure 2: 51.9 ± 10.2 CG: 55 ± 10.2
*Uncontrolled trials [n = 12]*									
Barrio et al. 2012^46^	Spain	49.1 ± 9.4	31 (100% female)	Breast cancer	Women affected by breast cancer	The Rosenberg Self-Esteem Scale	Self-esteem 1: 1.8 ± 0.6 Self-esteem 2: 1.9 ± 0.8		Self-esteem 1: 1.5 ± 0.6 Self-esteem 2: 1.5 ± 0.6
Caru et al. 2020^47^	Canada	12.1 ± 3.6	16 (50% female) N Total: 16 N Male:8 N Female: 8	Various cancer types	During cancer treatment	Self-esteem with the Physical Self-Perception Profile (PSPP)	Total: 5.3 ± 0.5 Male: 5.1 ± 0.4 Female: 5.4 ± 0.5		Total: 5.7 ± 0.5 Male: 5.8 ± 0.5 Female: 5.6 ± 0.5
Caru et al. 2021^48^	Canada	12.1 ± 3.6	16 (50% female) Boys: 8 Girls: 8	Various malignancy disease types	During cancer treatment	Self-esteem with the Physical Self-Perception Profile (PSPP)	Total: 5.8 ± 0.5 Boys: 5.6 ± 0.5 Girls: 5.9 ± 0.4		Total: 5.3 ± 0.5 Boys: 5.1 ± 0.4 Girls: 5.4 ± 0.5
Courneya et al. 2014^49^	Canada	˃ 18 years old	301 (100% female) Standard aerobic exercise: 96 High standard dose: 101 Combined physical activity: 104	Breast cancer	During cancer treatment	The Rosenberg Self-Esteem Scale	Standard aerobic exercise: 33.5 ± 4.3 High standard dose: 34.3 ± 5.2 Combined physical activity: 34.0 ± 5.2		Standard aerobic exercise: 34.8 ± 2.8 High standard dose: 34.5 ± 2.8 Combined physical activity: 33.9 ± 2.8
Ho, Rainbow et al. 2005^50^	Hong Kong	50.2 ± 7.1	Dance: 22	Various cancer types	During cancer treatment	The Rosenberg Self-Esteem Scale	Dance: 16.7 ± 3.3		Dance: 18.2 ± 3.6
Morielli et al. 2016^51^	Canada	57.5	18 (33.3% female) Total: 18	Rectal cancer	During cancer treatment	The Rosenberg Self-Esteem Scale	Total During NACRT^6^: 4.9 ± 1		Total Post-NACRT/pre-surgery: 4.8 ± 1
Muller et al. 2016^52^	Germany	10.7 ± 4.3	150 (49% female) Total: 150 N Leukemia/ lymphoma = 86 N Brain tumor = 38 N Sarcoma = 26	Various cancer types	After cancer treatment	Self-esteem with the KINDL questionnaire	Total: 67 ± 17.7		Total: 69 ± 17.1
Osypiuk et al. 2020^53^	USA	54 ± 10.2	21 (100% female) Qigong: 21	Breast cancer	After cancer treatment	The Rosenberg Self-Esteem Scale	Qigong :21.7 ± 6.1		Qigong: 23.7 ± 5.5
Rey-Barth et al. 2022^54^	France	52 (Range 46–55)	14 (100% female) Aerobic physical activity: 14	Breast cancer	After cancer treatment	The Rosenberg Self-Esteem Scale	Aerobic physical activity: 30.4 ± 6.6		Aerobic physical activity: 32.6 ± 5.5
Speed-Andrews et al. 2010^55^	Canada	54.8 ± 5.3	17 (100% female) Yoga: 17	Breast cancer	After cancer treatment	The Rosenberg Self-Esteem Scale	Yoga: 31.5 ± 5.7		Yoga: 33.8 ± 5.6
Török et al. 2006^56^	Hungary	15.6 ± 1.5	52 (57.7% female) Therapeutic recreation camping: 44	Various malignancy disease types	During cancer treatment	The Rosenberg Self-Esteem Scale	Therapeutic recreation camping: 27.2 ± 3.6		Therapeutic recreation camping: 28.3 ± 4.1
Vallet et al. 2015^57^	France	14.3 ± 2.9	11 (36.4% female) Combined physical activity: 11	Various cancer types	During cancer treatment	Self-esteem with the physical self-inventory (PSI-6)	Combined physical activity: 6.2 ± 2.1		Combined physical activity: 7.7 ± 1.8
*Observational studies [n = 5]*									
Awick et al. 2017^58^	USA	56.2 ± 9.3	370 (100% female) Total: 370	Breast cancer	After cancer treatment	The Rosenberg Self-Esteem Scale	Total: 40.5 ± 6		Total: 40.5 ± 5.6
Belanger et al. 2013^59^	Canada	38.2 ± 5.6	588 (43.7% female) No sport participation: 397 Sport participation: 191	Young adult cancer	After cancer treatment	The Rosenberg Self-Esteem Scale	No sport participation: 31.7 ± 5.8 Sport participation: 34.4 ± 4.8		N/A
Deisenroth et al. 2016^60^	Germany	11.4 ± 4.1	40 (57.5% female) Total: 40	Various cancer types	During cancer treatment	Self-esteem with the KINDL questionnaire	Total: 52.4 ± 20.9		N/A
Patsou et al. 2018^61^	Greece	51.7 ± 7.3	171 (100% male) Low fitness: 89 CG: 82	Breast cancer	After cancer treatment	Self-esteem with the Greek version of the Self-Esteem Scale	IG: 41.61 ± 3.30 CG: 32.67 ± 6.07		N/A
Ranft et al. 2017^62^	Germany	30 (Range 9–69)	909 (44.4% female) Survivors: 613 CG: 296	Ewing Sarcoma	After cancer treatment	The Rosenberg Self-Esteem Scale	Survivors: 23.2 CG: 24		N/A

Table 2 shows the characteristics of the interventions from studies included in the meta-analysis. Control groups received various interventions: usual care (73.3%)^24,25,34,36,37,39,42–45^, three educational sessions (13.3%)^35,38^, recreational activity (6.7%) ^23^, dietary guidelines and information about healthy habits (6.7%)^41^, and not have a control group (6.7%)^40^. PA interventions were categorized as follows: combined (i.e., aerobic + resistance PA) (38%) ^23,36,40–43,45^, aerobic (33%)^25,34,35,37,39,40,44^, mind-body (19%)^24,25,35,38^, and resistance (10%)^37,40^. Most interventions involved supervised exercises (69%), with the remainder either unsupervised (25%) or a combination of both (6%). The duration of the interventions ranged from 1 to 24 weeks (median = 13.4) with the weekly exercise duration of the intervention ranging from 45 to 330 min. Characteristics of intervention studies not included in the meta-analysis are detailed in Table S5.

**Table 2:** Characteristic of studies’ interventions included in the meta-analysis.

Reference	Control group	Intervention type	Categorization	Duration (weeks)	Volume (minutes per week)	Supervision
*Randomized Controlled Trials [n = 13]*						
Adams et al. 2018^34^	Received usual care	Uphill treadmill walking or running, and to maintain all other exercise they were performing at baseline	Aerobic physical activity	12 weeks	180 min	Yes
Boing et al. 2023^35^	Received an invitation to three educational sessions	IG 1: Pilates IG 2: Belly dance	IG 1: Mind-body exercise IG 2: Aerobic physical activity	16 weeks	180 min	Yes
Cadmus et al. 2009^1,^^36^	Received usual care	Variety of sports/recreational activities	Combined physical activity	24 weeks	150 min	Yes
Cadmus et al. 2009^2,^^36^	Received usual care	Variety of sports/recreational activities	Combined physical activity	24 weeks	150 min	Yes
Courneya et al. 2007^37^	Received usual care	IG 1: Aerobic physical activity IG 2: Resistance training	IG 1: Aerobic physical activity IG 2: Resistance training	17 weeks	> 135 min	Yes
Fretta et al. 2021^38^	Three educational sessions	Pilates method intervention	Mind-body exercise	16 weeks	180 min	Yes
Gokal et al. 2016^39^	Received usual care	Moderate intensity walking	Aerobic physical activity	12 weeks	About 150 min	No
Kovačič et al. 2011^24^	Received usual care	Relaxation training sessions according to the Yoga in Daily Life system.	Mind-body exercise	3 weeks	105 min	No
Leite et al. 2021^25^	Received usual care	IG 1: Belly dance IG 2: Mat Pilates	IG 1: Aerobic physical activity IG 2: Mind-body exercise	16 weeks	180 min	Yes
Musanti 2012^40^	No CG (Participant divided in Aerobic, Resistance, Combined and flexibility*)	IG 1: Aerobic physical activity IG 2: Resistance training IG 3: Aerobic + Resistance training	IG 1: Aerobic physical activity IG 2: Resistance training IG 3: Combined physical activity	12 weeks	45–90 min	No
Rastogi et al. 2020^41^	Received Dietary Guidelines, standardized e-mails at 1, 2, 4, and 8 weeks with information on healthy eating and stress management	Multi-component intervention	Combined physical activity	12 weeks	170 ± 131 min	No
Saultier et al. 2021^23^	Received recreational activities the first 6 month and later do the physical activity program of 6 month	Strength and muscle building, balance and proprioception training and 15 multi-activity sessions (dance, basketball, badminton, yoga, skiing, swimming, paddling, etc.).	Combined physical activity	24 weeks	120–330 min	Yes
Van Dijk-Lokkart et al. 2016^42^	Received usual care	Cardiorespiratory and muscle strength training	Combined physical activity	12 weeks	90 min	Yes
Wurz et al. 2019^43^	Received usual care	Aerobic and strength training sessions	Combined physical activity	12 weeks	100–180 min	Mixed
*Quasi-Experimental studies [n = 2]*						
Carminatti et al. 2019^44^	Received usual care	Belly dance	Aerobic physical activity	12 weeks	120 min	Yes
Rosenberg et al. 2014^45^	Received usual care	IG 1: Outdoor adventure program 1 IG 2: Outdoor adventure program 2	Combined physical activity	1 week	-	Yes

Additional information of the intervention studies not included in the meta-analysis can be found in the supplementary material Table S5. *IG* Intervention group, *CG* Control group

### Meta-analysis

**Figure 2:**
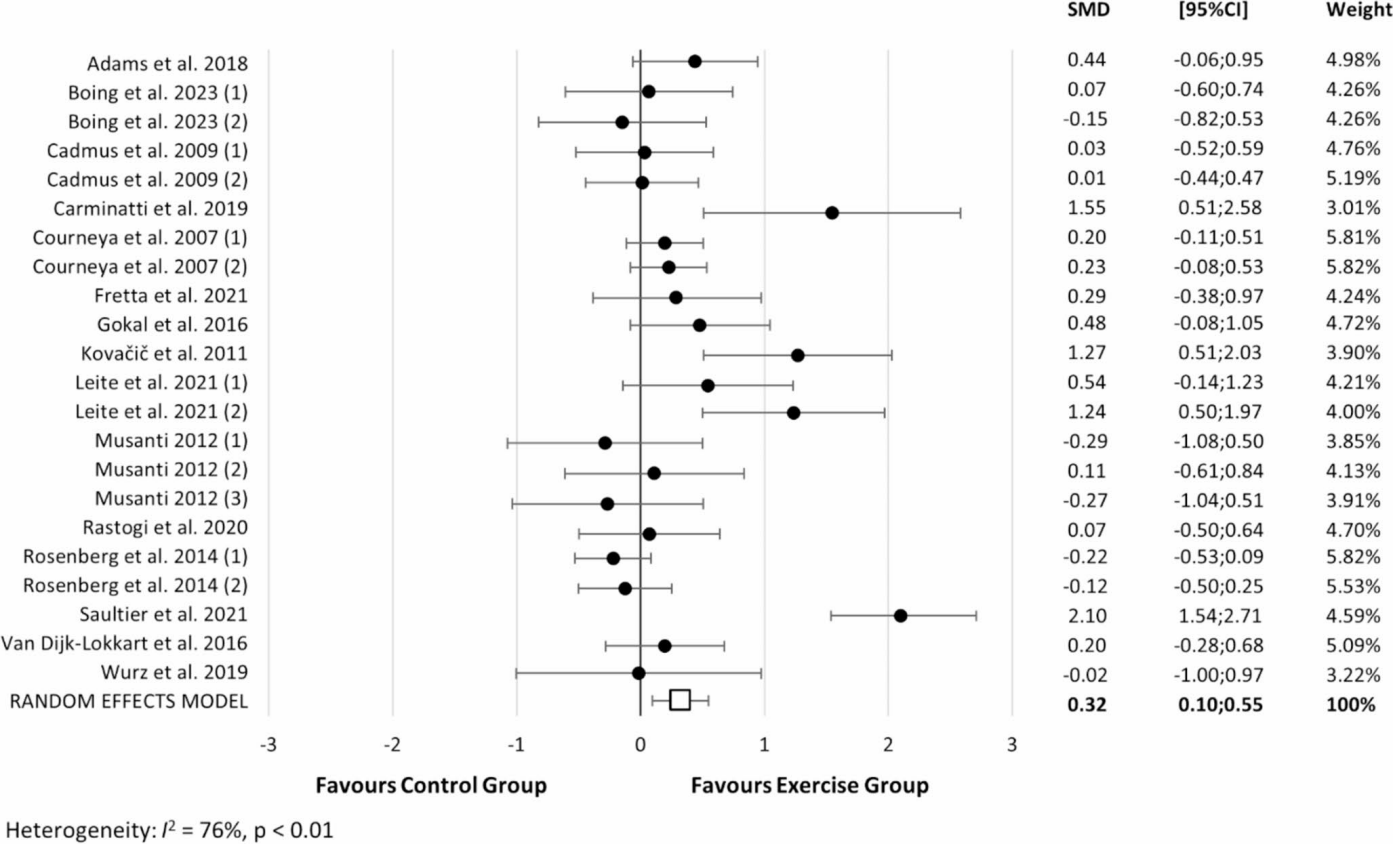
Forest plot of overall physical activity interventions on self-esteem during and after cancer treatment. SMD: Standardized mean difference; CI: confidence intervals. Boing et al. 2023 (1): represents the mind-body exercise; Boing et al. 2023 (2): aerobic physical activity; Cadmus et al. 2009 (1): combined physical activity during cancer treatment; Cadmus et al. 2009 (2): combined physical activity after cancer treatment; Courneya et al. 2007 (1): aerobic physical activity; Courneya et al. 2007 (2): resistance training; Leite et al. 2021 (1): aerobic physical activity; Leite et al. 2021 (2): mind-body exercise; Musanti 2012 (1): aerobic physical activity; Musanti 2012 (2): resistance training; Musanti 2012 (3): combined physical activity; Rosenberg et al. 2014 (1): outdoor adventure 1 (people for whom it was their first outdoor adventure program); Rosenberg et al. 2014 (2): outdoor adventure 2: people for whom it was their second outdoor adventure program.

A total of 15 studies examining the effect of PA intervention with a control group on self-esteem during (36.4%), after (54.5%), and both during and after cancer treatment (9.1%) were included in this meta-analysis. The pooled SMD of all PA interventions on self-esteem was 0.32 (95% CI: 0.10 to 0.55, *p* < 0.01, I^2^ = 76%) for changes in self-esteem across all types of exercise (Fig. 2). There was no statistically significant publication bias according to Egger’s test (*P* = 0.097) or based on a visual inspection of the funnel plot for self-esteem outcome *(Supplementary figure **S1**).* However, after incorporating imputed studies (*N* = 3) using the “trim and fill” procedure, the SMD estimate was 0.418 (95% CI: 0.186 to 0.650). Thus, correction for potential publication bias did not alter the significance of the results.

**Figure 3:**
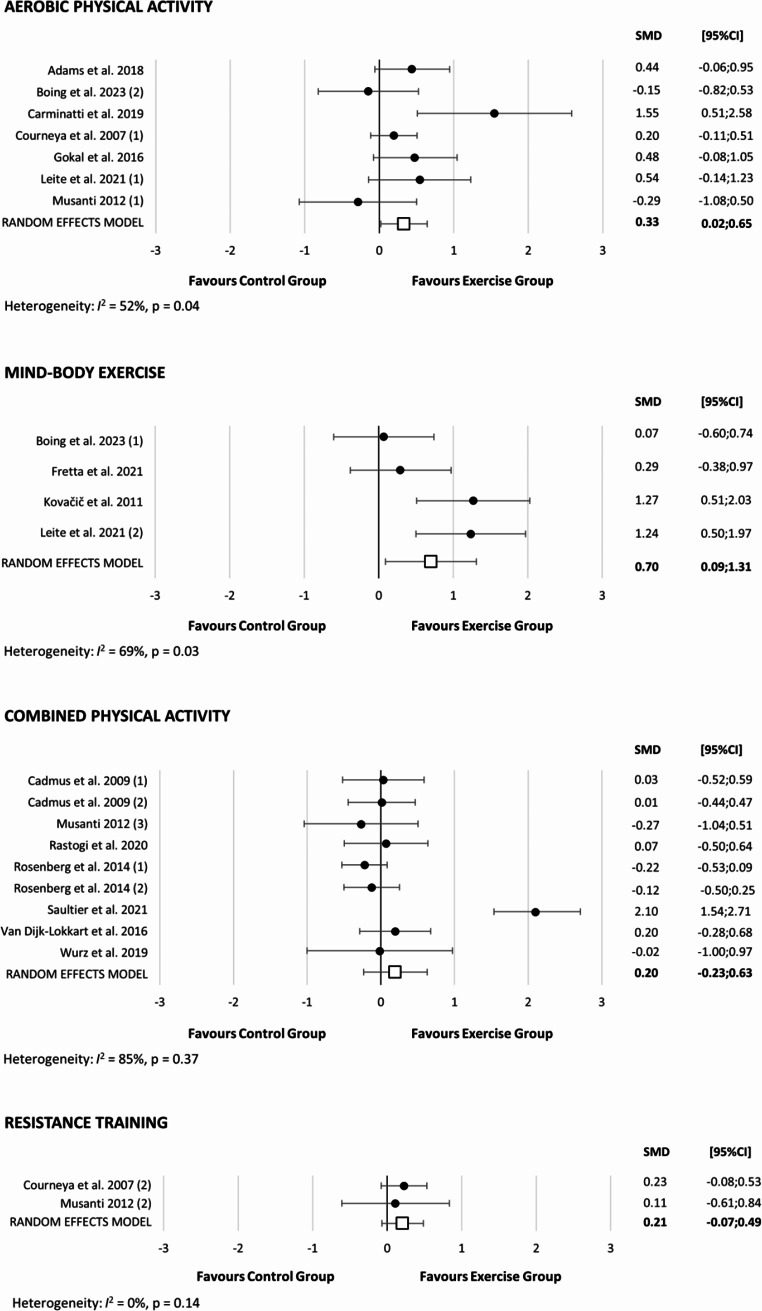
Forest plot of physical activity interventions divided by its type on self-esteem during and after cancer treatment. SMD: Standardized mean difference; CI: confidence intervals. Boing et al. 2023 (1): represents the mind-body exercise; Boing et al. 2023 (2): aerobic physical activity; Cadmus et al. 2009 (1): combined physical activity during cancer treatment; Cadmus et al. 2009 (2): combined physical activity after cancer treatment; Courneya et al. 2007 (1): aerobic physical activity; Courneya et al. 2007 (2): resistance training; Leite et al. 2021 (1): aerobic physical activity; Leite et al. 2021 (2): mind-body exercise; Musanti 2012 (1): aerobic physical activity; Musanti 2012 (2): resistance training; Musanti 2012 (3): combined physical activity; Rosenberg et al. 2014 (1): outdoor adventure 1 (people for whom it was their first outdoor adventure program); Rosenberg et al. 2014 (2): outdoor adventure 2: people for whom it was their second outdoor adventure program.

Exploratory subgroup analyses were conducted to assess changes in self-esteem based on the type of PA intervention (Fig. 3). For aerobic PA interventions, the SMD was 0.33 (95% CI: -0.02 to 0.65, *p* = 0.04, I^2^ = 52%). Mind-body exercise interventions showed a larger SMD of 0.70 (95% CI: 0.09 to 1.31, *p* = 0.03, I^2^ = 69%). The ‘trim and fill’ procedure for this analysis indicated no changes in estimates, and no correction for potential publication bias was needed (data not shown). Similarly, the leave-one-out analysis did not alter the results (data not shown). For combined PA interventions, the SMD was 0.20 (95% CI: -0.23 to 0.63, *p* = 0.37, I^2^ = 85%). Given the limited number of studies examining resistance training interventions on self-esteem (*n* = 2), the SMD appeared to align with that of combined PA interventions (SMD = 0.21, 95% CI: -0.07 to 0.49, *p* = 0.14, I^2^ = 0%). The leave-one-out analysis for these exploratory subgroup analyses did not alter the results (data not shown).

Regarding the effects of overall PA interventions on self-esteem considering cancer status (during vs. after cancer treatment) and the length of the intervention (12 weeks or less vs. more than 12 weeks), for patients during cancer treatment, the SMD was 0.50 (95% CI: 0.11 to 0.89, *p* = 0.01, I^2^ = 87%), whereas for those after cancer treatment, the SMD was 0.09 (95% CI: -0.10 to 0.29, *p* = 0.35, I^2^ = 40%) (*Supplementary figure S2*). Additionally, interventions lasting 12 weeks or less had an SMD of 0.21 (95% CI: -0.06 to 0.48, *p* = 0.13, I^2^ = 64%), while those lasting more than 12 weeks showed a higher SMD of 0.44 (95% CI: 0.06 to 0.82, *p* = 0.02, I^2^ = 82%) *(Supplementary figure S3)*. The leave-one-out analysis for these exploratory analyses did not alter the results (data not shown).

When examining the exploratory subgroup analyses across groups of age, study design, and self-esteem questionnaire, the limited number of studies makes it difficult to draw any definitive conclusions. For children and adolescents, the SMD was 1.15 (95% CI: -0.74 to 3.04, *p* = 0.23, I^2^ = 96%), while for adults the SMD was 0.22 (95% CI: 0.04 to 0.40, *p* = 0.02, I^2^ = 56%) (*Supplementary Figure S4*). For quasi-experimental studies the SMD was 0.21 (95% CI: -0.44 to 0.86, *p* = 0.53, I^2^ = 83%) whereas for randomized controlled trial the SMD was 0.35 (95% CI: 0.11 to 0.59, *p* < 0.01, I^2^ = 72%) (*Supplementary Figure S5*). For questionnaires other than the Rosenberg Self-Esteem Scale, the SMD was 0.47 (95% CI: -0.40 to 1.34, *p* = 0.29, I^2^ = 94%) while for the Rosenberg self-esteem scale the SMD was 0.28 (95% CI: 0.10 to 0.47, *p* < 0.01, I^2^ = 48%) (*Supplementary Figure S6*).

## Discussion

To our knowledge, this is the first systematic review and meta-analysis to focus on the effects of PA on self-esteem during and after cancer treatment. Our findings suggest that PA interventions have a small but positive effect on self-esteem in this population. Specifically, aerobic PA showed a small positive effect on self-esteem, while mind-body exercise showed a medium positive effect. However, no significant effects were observed for combined PA or resistance training on self-esteem. Regarding the interventions conducted during cancer treatments, as well as those lasting more than 12 weeks, it had a positive effect on self-esteem, with medium and small effect, respectively. No significant effects were found in additional analyses in group of age, study design and self-esteem questionnaires.

Our findings indicate that aerobic PA interventions improved self-esteem during cancer treatment, but not after cancer treatment. The study by Carminatti et al.^44^ notably contributed to these results, although some studies showed trends towards significance ^25,34,39^. In the studies by Carminatti et al.^44^, Boing et al.^35^, and Leite et al.^25^, belly dance interventions were used for women with breast cancer during and after cancer treatment. However, only Carminatti et al. reported significant improvements in self-esteem. One possible explanation for these differing results is that participants in the studies by Boing et al. and Leite et al. reported higher baseline self-esteem scores compared than those in Carminatti et al., suggesting the latter group may had more room for improvement. In addition, the use of a mirror during Carminatti et al., intervention may have played a role in enhancing self-esteem, as the authors noted that mirrors may help participants refine technique and posture, fostering greater confidence and self-esteem^35^. Other studies employed treadmill, elliptical, or moderate-intensity walking interventions, such as Courneya et al.^37^ and Musanti et al.^40^ after breast cancer treatment, Gokal et al.^39^ during breast cancer, and Adams et al.^34^ after testicular cancer treatment. Of these, only Gokal et al.^39^ and Adams et al.^34^ reported results tending towards significance. These findings may be influenced by higher baseline self-esteem in the control group, except Gokal et al.^39^. Moreover, the authors suggest that the intensity and duration of the interventions might have been insufficient to yield significant improvements.

For mind-body exercise interventions, our results suggest a positive effect on improving self-esteem during and after cancer treatment. Supporting this, a study on university students found a positive relationship between a Yoga Nidra intervention and self-esteem^63^. The authors of the study attribute this effect to the relaxation mechanisms of the intervention, which may increase parasympathetic system activity, reducing psychological stress and, in turn, enhancing self-esteem ^63^. Additionally, most of the articles in this meta-analysis (75%) featured interventions lasting than 12 weeks, which may further explain the positive effect of mind-body exercise on self-esteem in this population.

Finally, our analysis found no significant effect of combined PA and resistance training interventions on self-esteem during and after cancer treatment. Several factors may explain these results. First, three of the interventions were home-based, which limited social interaction. Second, only 28.6% of the interventions lasted longer than 12 weeks, which may be insufficient time to see a significant effect. Third, many interventions allocated more time to aerobic PA than resistance training, and most studies (75%) focused on individuals after cancer treatment. Regarding resistance training, the limited number of studies and small sample sizes reduce the statistical power, making it difficult to determine whether this type of intervention has a positive impact on self-esteem.

Our exploratory subgroup analyses identified two key factors: cancer status (during cancer treatment) and intervention duration (over 12 weeks), that contributed to the effects of PA on self-esteem. Firstly, a stress response is common after a cancer diagnosis and usually decreases over time^64^. However, prolonged stress can lead to chronic issues that require professional intervention^64,65^. This suggests that individuals after cancer treatment who are highly stressed and not fully recovered may need more than just PA to improve self-esteem; psychological support may be necessary. Secondly, regular PA boosts the production and release of brain-derived neurotrophic factor (BDNF)^66^, a vital protein for the central nervous system that supports synaptic formation, maintenance, and neuroplasticity^67^. Increased BDNF levels are linked to enhanced cognitive function and emotional well-being^68^. A meta-analysis on exercise and depression found that the most significant improvements occurred around the 16-week mark ^69^. Given the strong connection between depression and self-esteem^70^, this could explain why longer PA interventions have a more pronounced positive effect on self-esteem. In relation to the additional exploratory subgroup analyses in group of age, study design, and self-esteem questionnaire, along with the analysis of resistance training interventions, it is difficult to draw a conclusion due to the limited number of studies in these conditions.

### Strengths and limitations

This systematic review and meta-analysis provide a thorough qualitative and quantitative assessment of PA interventions and their effects on self-esteem during and after cancer treatment. However, several limitations should be noted. First, the limited number of studies focusing on the paediatric population prevents us from drawing robust conclusions for this specific group. Second, the findings should be interpreted with caution due to the overall limited number of studies on this topic and the lack of evidence regarding the safety of PA during cancer treatments. Third, high levels of heterogeneity among studies necessitate careful interpretation of the results. Fourth, some studies could not be included in the analysis due to inaccessible full-text articles and a lack of response from authors when contacted.

### Conclusion

Our systematic review and meta-analysis indicate that PA (primarily aerobic and mind-body exercise) may enhance self-esteem during and after cancer treatment. Additionally, the cancer status and duration of the intervention appear to significantly influence the impact of PA on self-esteem.

## Critical view

Psychological factors, including altered levels of self-esteem, are among the most common causes of cancer and its treatment. While previous systematic reviews and meta-analyses have shown that different types of PA reduce depression, anxiety, and fatigue during and after cancer treatment, the impact of PA on self-esteem has been less thoroughly investigated. This systematic review and meta-analysis may help existing research on this topic, revealing that PA interventions, particularly aerobic and mind-body exercise, may enhance self-esteem both during and after cancer treatment. Additionally, factors such as the cancer status (i.e., individuals during cancer treatment) and the duration of the intervention (more than 12 weeks) significantly influence the effectiveness of PA on self-esteem.

## Supplementary Information

Below is the link to the electronic supplementary material.

Supplementary Material 1

## Data Availability

The data that support the findings of this study are available from the corresponding author upon reasonable request.
