# Supplementary material for: The effects of physical activity interventions on self-esteem during and after cancer treatment: a systematic review and meta-analysis
Source: Sci Rep. 2024 Nov 6;14:26849. doi: 10.1038/s41598-024-74888-2 (PMC11538342; doi:10.1038/s41598-024-74888-2)
Supplement: Supplementary file 1 — Supplementary Material 1 [file 41598_2024_74888_MOESM1_ESM.docx]

| **Supplementary table S1. PRISMA-S 2021 Checklist** | | | |
| --- | --- | --- | --- |
| **Section and Topic** | **Item #** | **Checklist item** | **Location where item is reported** |
| **INFORMATION SOURCES AND METHODS** | | | |
| Database name | 1 | Name each individual database searched, stating the platform for each. | Page 4 |
| Multi-database searching | 2 | If databases were searched simultaneously on a single platform, state the name of the platform, listing all of the databases searched. | No applicable |
| Study registries | 3 | List any study registries searched. | No applicable |
| Online resources and browsing | 4 | Describe any online or print source purposefully searched or browsed (e.g., tables of contents, print conference proceedings, web sites), and how this was done. | No applicable |
| Citation searching | 5 | Indicate whether cited references or citing references were examined, and describe any methods used for locating cited/citing references (e.g., browsing reference lists, using a citation index, setting up email alerts for references citing included studies). | Page 5 |
| Contacts | 6 | Indicate whether additional studies or data were sought by contacting authors, experts, manufacturers, or others. | Page 5 |
| Other methods | 7 | Describe any additional information sources or search methods used. | Page 5 |
| **SEARCH STRATEGIES** | | | |
| Full search strategies | 8 | Include the search strategies for each database and information source, copied and pasted exactly as run. | Supplementary table S2 |
| Limits and restrictions | 9 | Specify that no limits were used, or describe any limits or restrictions applied to a search (e.g., date or time period, language, study design) and provide justification for their use. | Page 4 |
| Search filters | 10 | Indicate whether published search filters were used (as originally designed or modified), and if so, cite the filter(s) used. | No |
| Prior work | 11 | Indicate when search strategies from other literature reviews were adapted or reused for a substantive part or all of the search, citing the previous review(s). | Page 4 |
| Updates | 12 | Report the methods used to update the search(es) (e.g., rerunning searches, email alerts). | Page 4 |
| Dates of searches | 13 | For each search strategy, provide the date when the last search occurred. | All the search occurred in the same moment (Page 4) |
| **PEER REVIEW** | | | |
| Peer review | 14 | Describe any search peer review process. | Page 4 and 5 |
| **MANAGING RECORDS** | | | |
| Total Records | 15 | Document the total number of records identified from each database and other information sources. | Figure 1 |
| Deduplication | 16 | Describe the processes and any software used to deduplicate records from multiple database searches and other information sources. | Page 5 and 6 |

Table S1 show the PRISMA-S 2021 Checklist

| **Supplementary table S2. PRISMA 2020 Checklist** | | | |
| --- | --- | --- | --- |
| **Section and Topic** | **Item #** | **Checklist item** | **Location where item is reported** |
| **TITLE** | | |  |
| Title | 1 | Identify the report as a systematic review. | Page 1 |
| **ABSTRACT** | | |  |
| Abstract | 2 | See the PRISMA 2020 for Abstracts checklist. | Page 1-2 |
| **INTRODUCTION** | | |  |
| Rationale | 3 | Describe the rationale for the review in the context of existing knowledge. | Page 5 |
| Objectives | 4 | Provide an explicit statement of the objective(s) or question(s) the review addresses. | Page 5 |
| **METHODS** | | |  |
| Eligibility criteria | 5 | Specify the inclusion and exclusion criteria for the review and how studies were grouped for the syntheses. | Page 6 |
| Information sources | 6 | Specify all databases, registers, websites, organisations, reference lists and other sources searched or consulted to identify studies. Specify the date when each source was last searched or consulted. | Page 6 |
| Search strategy | 7 | Present the full search strategies for all databases, registers and websites, including any filters and limits used. | Supplementary Table 2 |
| Selection process | 8 | Specify the methods used to decide whether a study met the inclusion criteria of the review, including how many reviewers screened each record and each report retrieved, whether they worked independently, and if applicable, details of automation tools used in the process. | Page 6-7 |
| Data collection process | 9 | Specify the methods used to collect data from reports, including how many reviewers collected data from each report, whether they worked independently, any processes for obtaining or confirming data from study investigators, and if applicable, details of automation tools used in the process. | Page 6-7 |
| Data items | 10a | List and define all outcomes for which data were sought. Specify whether all results that were compatible with each outcome domain in each study were sought (e.g. for all measures, time points, analyses), and if not, the methods used to decide which results to collect. | Page 6-7 |
|  | 10b | List and define all other variables for which data were sought (e.g. participant and intervention characteristics, funding sources). Describe any assumptions made about any missing or unclear information. | Page 6 and Table 1 |
| Study risk of bias assessment | 11 | Specify the methods used to assess risk of bias in the included studies, including details of the tool(s) used, how many reviewers assessed each study and whether they worked independently, and if applicable, details of automation tools used in the process. | Page 6-7 |
| Effect measures | 12 | Specify for each outcome the effect measure(s) (e.g. risk ratio, mean difference) used in the synthesis or presentation of results. | Table 1 |
| Synthesis methods | 13a | Describe the processes used to decide which studies were eligible for each synthesis (e.g. tabulating the study intervention characteristics and comparing against the planned groups for each synthesis (item #5)). | Page 6-7 |
|  | 13b | Describe any methods required to prepare the data for presentation or synthesis, such as handling of missing summary statistics, or data conversions. | Page 7-8 |
|  | 13c | Describe any methods used to tabulate or visually display results of individual studies and syntheses. | Page 7-8 |
|  | 13d | Describe any methods used to synthesize results and provide a rationale for the choice(s). If meta-analysis was performed, describe the model(s), method(s) to identify the presence and extent of statistical heterogeneity, and software package(s) used. | Page 7-8 |
|  | 13e | Describe any methods used to explore possible causes of heterogeneity among study results (e.g. subgroup analysis, meta-regression). | Page 7-8 |
|  | 13f | Describe any sensitivity analyses conducted to assess robustness of the synthesized results. | Page 7-8 |
| Reporting bias assessment | 14 | Describe any methods used to assess risk of bias due to missing results in a synthesis (arising from reporting biases). | Page 7-8 |
| Certainty assessment | 15 | Describe any methods used to assess certainty (or confidence) in the body of evidence for an outcome. | Page 7-8 |
| **RESULTS** | | |  |
| Study selection | 16a | Describe the results of the search and selection process, from the number of records identified in the search to the number of studies included in the review, ideally using a flow diagram. | Page 8 and Figure 1 |
|  | 16b | Cite studies that might appear to meet the inclusion criteria, but which were excluded, and explain why they were excluded. | Figure 1 |
| Study characteristics | 17 | Cite each included study and present its characteristics. | Page 9 and Table 1, Table 2 and Table S4 |
| Risk of bias in studies | 18 | Present assessments of risk of bias for each included study. | Page 8 and Table S3 |
| Results of individual studies | 19 | For all outcomes, present, for each study: (a) summary statistics for each group (where appropriate) and (b) an effect estimate and its precision (e.g. confidence/credible interval), ideally using structured tables or plots. | Page 9-10 and Figure 2 and Figure 3 |
| Results of syntheses | 20a | For each synthesis, briefly summarise the characteristics and risk of bias among contributing studies. | Page 8 and Table S3 |
|  | 20b | Present results of all statistical syntheses conducted. If meta-analysis was done, present for each the summary estimate and its precision (e.g. confidence/credible interval) and measures of statistical heterogeneity. If comparing groups, describe the direction of the effect. | Page 9-10 and Figure 2 and Figure 3 |
|  | 20c | Present results of all investigations of possible causes of heterogeneity among study results. | Page 9-10 |
|  | 20d | Present results of all sensitivity analyses conducted to assess the robustness of the synthesized results. | Page 9-10 |
| Reporting biases | 21 | Present assessments of risk of bias due to missing results (arising from reporting biases) for each synthesis assessed. | Page 8 and Table S3 |
| Certainty of evidence | 22 | Present assessments of certainty (or confidence) in the body of evidence for each outcome assessed. | Page 8 |
| **DISCUSSION** | | |  |
| Discussion | 23a | Provide a general interpretation of the results in the context of other evidence. | Paged 10-12 |
|  | 23b | Discuss any limitations of the evidence included in the review. | Page 12 |
|  | 23c | Discuss any limitations of the review processes used. | Page 12 |
|  | 23d | Discuss implications of the results for practice, policy, and future research. | Page 12 |
| **OTHER INFORMATION** | | |  |
| Registration and protocol | 24a | Provide registration information for the review, including register name and registration number, or state that the review was not registered. | Page 5-6 |
|  | 24b | Indicate where the review protocol can be accessed, or state that a protocol was not prepared. | Page 5-6 |
|  | 24c | Describe and explain any amendments to information provided at registration or in the protocol. | Page 5-6 |
| Support | 25 | Describe sources of financial or non-financial support for the review, and the role of the funders or sponsors in the review. | Yes. Page 3 |
| Competing interests | 26 | Declare any competing interests of review authors. | No |
| Availability of data, code and other materials | 27 | Report which of the following are publicly available and where they can be found: template data collection forms; data extracted from included studies; data used for all analyses; analytic code; any other materials used in the review. | No applicable |

Table S2 show the PRISMA 2020 Checklist

| **Supplementary table S3**. Search terms used in databases. |
| --- |
| **MEDLINE (via PubMed)** |
| TITLE/ABSTRACT ("physical conditioning" OR "motor activity" OR "physical activity" OR "activity" OR "physical education" OR exercis* OR move* OR moving OR active OR inactive OR sedentary OR sport* OR train*) AND ("psychological wellbeing" OR "psychological well-being" OR "self-esteem" OR "self-concept" OR "self-worth") AND (cancer OR onco* OR myelo* OR leukaemia OR leukemia OR neoplasm* OR lympho* OR carcinoma OR tumor OR tumour OR sarcoma) |
| **Scopus** |
| TITLE-ABS-KEY ( ( "physical conditioning" OR "motor activity" OR "physical activity" OR "activity" OR "physical education" OR exercis* OR move* OR moving OR active OR inactive OR sedentary OR sport* OR train* ) AND ( "psychological wellbeing" OR "psychological well-being" OR "self-esteem" OR "self-concept" OR "self-worth" ) AND ( cancer OR onco* OR myelo* OR leukaemia OR leukemia OR neoplasm* OR lympho* OR carcinoma OR tumor OR tumour OR sarcoma ) ) |
| **SPORTDiscuss** |
| ABSTRACT ("physical conditioning" OR "motor activity" OR "physical activity" OR "activity" OR "physical education" OR exercis* OR move* OR moving OR active OR inactive OR sedentary OR sport* OR train*) AND ("psychological wellbeing" OR "psychological well-being" OR "self-esteem" OR "self-concept" OR "self-worth") AND (cancer OR onco* OR myelo* OR leukaemia OR leukemia OR neoplasm* OR lympho* OR carcinoma OR tumor OR tumour OR sarcoma) |
| **Psycinfo** |
| TITLE/ABSTRACT ("physical conditioning" OR "motor activity" OR "physical activity" OR "activity" OR "physical education" OR exercis* OR move* OR moving OR active OR inactive OR sedentary OR sport* OR train*) AND ("psychological wellbeing" OR "psychological well-being" OR "self-esteem" OR "self-concept" OR "self-worth") AND (cancer OR onco* OR myelo* OR leukaemia OR leukemia OR neoplasm* OR lympho* OR carcinoma OR tumor OR tumour OR sarcoma) |
| **Web of Science** |
| ABSTRACT ("physical conditioning" OR "motor activity" OR "physical activity" OR "activity" OR "physical education" OR exercis* OR move* OR moving OR active OR inactive OR sedentary OR sport* OR train*) AND ("psychological wellbeing" OR "psychological well-being" OR "self-esteem" OR "self-concept" OR "self-worth") AND (cancer OR onco* OR myelo* OR leukaemia OR leukemia OR neoplasm* OR lympho* OR carcinoma OR tumor OR tumour OR sarcoma) |
| Supplementary table S3 show the search terms used in databases |

| **Supplementary table S4**. Risk-of Bias Assessment of studies included in the meta-analysis | | | | | | | | | | | | | | | |
| --- | --- | --- | --- | --- | --- | --- | --- | --- | --- | --- | --- | --- | --- | --- | --- |
| 1. Cochrane Collaboration’s tool for assessing risk of bias (RoB2) for randomized controlled trials | | | | | | | | | | | | | | | |
|  | Randomization process | | Deviations from intended interventions | | | Missing outcome data | | Measurement of the outcome | | | Selection of the reported result | **Overall Bias** | | | |
| Boing et al. 2023 | 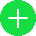 | | 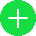 | | | 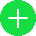 | | 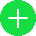 | | | 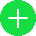 | 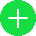 | | | |
| Cadmus et al. 2009 | 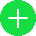 | | 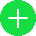 | | | 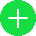 | | 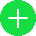 | | | 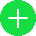 | 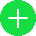 | | | |
| Courneya et al. 2007 | 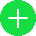 | | 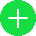 | | | 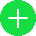 | | 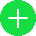 | | | 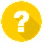 | 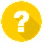 | | | |
| De Bem Fretta et al. 2021 | 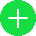 | | 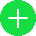 | | | 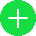 | | 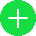 | | | 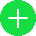 | 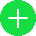 | | | |
| Gokal et al. 2016 | 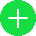 | | 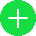 | | | 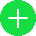 | | 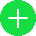 | | | 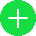 | 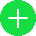 | | | |
| Kovacic et al. 2011 | 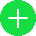 | | 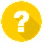 | | | 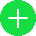 | | 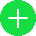 | | | 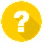 | 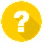 | | | |
| Leite et al. 2021 | 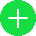 | | 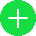 | | | 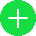 | | 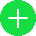 | | | 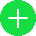 | 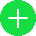 | | | |
| Musanti 2012 | 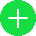 | | 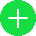 | | | 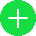 | | 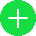 | | | 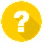 | 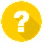 | | | |
| Rastogi et al. 2020 | 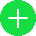 | | 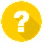 | | | 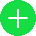 | | 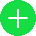 | | | 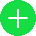 | 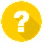 | | | |
| Saultier et al. 2021 | 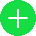 | | 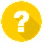 | | | 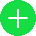 | | 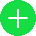 | | | 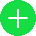 | 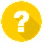 | | | |
| Scot et al. 2018 | 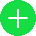 | | 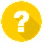 | | | 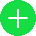 | | 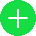 | | | 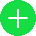 | 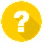 | | | |
| Van Dijk-Lokkart et al. 2016 | 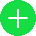 | | 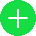 | | | 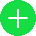 | | 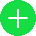 | | | 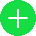 | 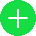 | | | |
| Wurz et al. 2019 | 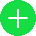 | | 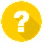 | | | 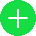 | | 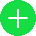 | | | 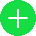 | 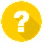 | | | |
| 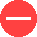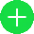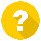Low risk Some concerns High risk | | | | | | | | | | | | | | | |
| 1. Joanna Briggs Institute critical appraisal tools for quasi-Experimental Studies | | | | | | | | | | | | | | | |
|  | Cause-effect | Similar comparison groups | | Receiving similar treatment/care | Control group | | Multiple measurements of the outcome | | Follow-up | Measured in the same way | | Measured in a reliable way | Statistical analysis | Quality category |  |
| Carminatti, M. et al. 2019 | ✓ | **×** | | ✓ | ✓ | | ✓ | | ? | ✓ | | ✓ | ✓ | High |  |
| Rosenberg R. et al. 2014 | ✓ | ? | | ✓ | ✓ | | ✓ | | ✓ | ✓ | | ✓ | ✓ | High |  |
| ✓: meet the methodological quality criterion; 🗶: not meet the methodological quality criterion.; ?: unclear. | | | | | | | | | | | | | | | |

Supplementary table S4 show the risk-of Bias Assessment of studies included in the meta-analysis (both Randomized Controlled Trials and quasi-Experimental Studies)

| **Supplementary table S5**. Characteristics of studies’ interventions not included in the meta-analysis. | | | | | | |
| --- | --- | --- | --- | --- | --- | --- |
| **Reference** | **Control group** | **Exclusion reason** | **Intervention type** | **Duration**  **(weeks)** | **Volume (minutes per week)** | **Supervision**  **of intervention**  **program** |
| *Uncontrolled trials [n=12]* |  |  |  |  |  |  |
| Caru et al. 2020 | No | No control group | Combined physical activity | 6 weeks | 180 minutes | Yes |
| Muller et al. 2016 | No | No control group | Combined physical activity | 4 weeks | 70 minutes | Yes |
| Vallet et al. 2015 | No | No control group | Combined physical activity | 6 weeks | 60-120 minutes | Yes |
| Barrio et al. 2012 | No | No control group | Combined physical activity | 12 weeks | 180 minutes | Yes |
| Morielli et al. 2016 | No | No control group | Aerobic physical activity | 6 weeks | 150 minutes | Mixed |
| Courneya et al. 2014 | No | No control group | Combined physical activity | Aprox 16 weeks | STAN: 75 minutes  HIGH: 150 minutes COMB: 150-180 minutes | Yes |
| Osypiuk et al. 2020 | No | No control group | Mind-body exercise | 12 weeks | 180-240 minutes | Yes and No |
| Ho, Rainbow et al. 2005 | No | No control group | Aerobic physical activity | 6 weeks | 90 minutes | Yes |
| Caru et al. 2021 | No | No control group | Combined physical activity | 6 weeks | - | - |
| Rey-Barth et al. 2022 | No | No control group | Aerobic physical activity | 6 weeks | 60-120 minutes | Yes |
| Török et al. 2006 | No | No control group | Therapeutic recreation camping | 8 weeks | - | - |
| Speed-Andrews et al. 2010 | No | No control group | Mind-body exercise | 12 weeks | 180 minutes | Yes |

Supplementary table S5 show the characteristic of the intervention of the studies not included in the meta-analysis.

**
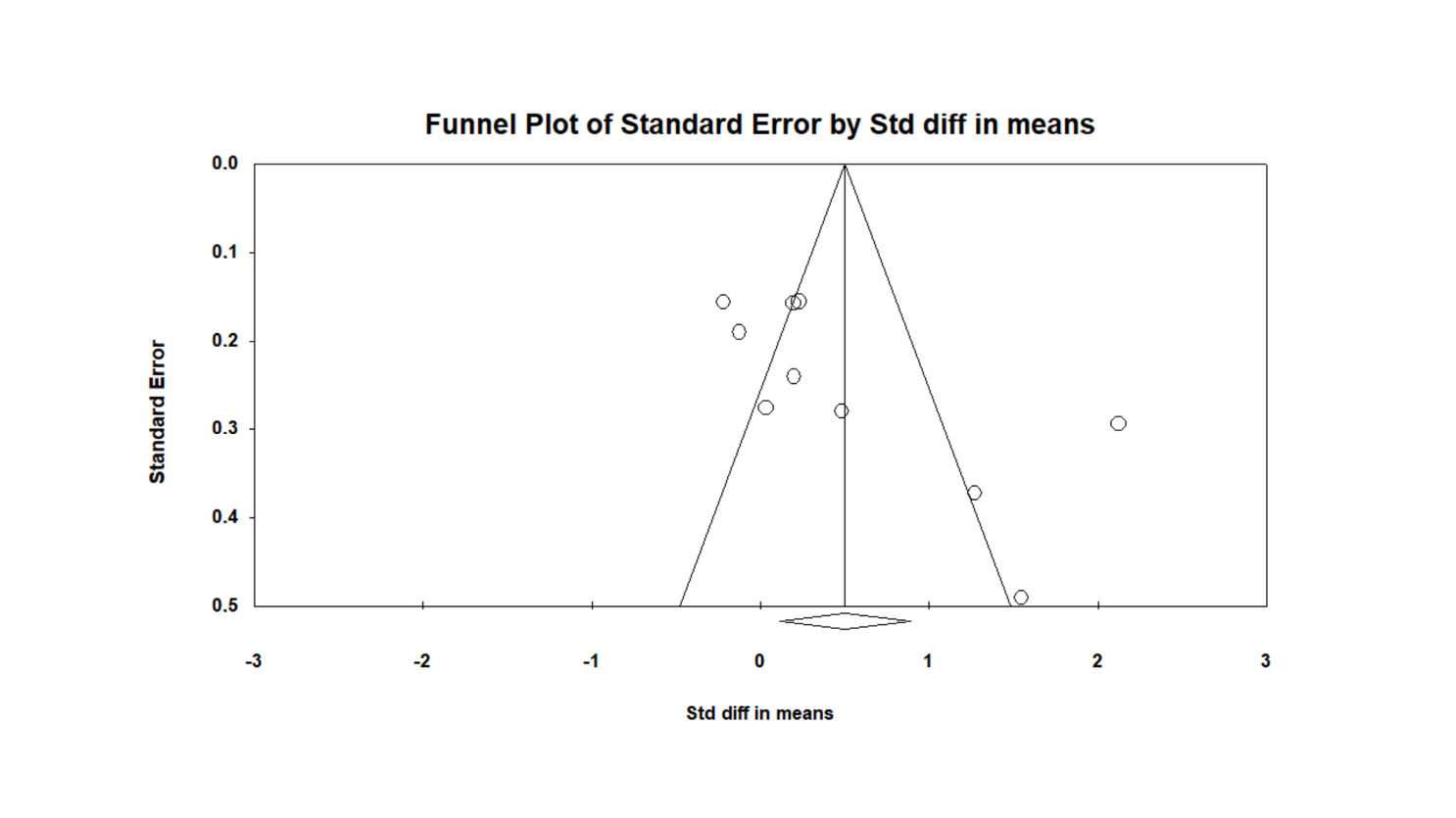
**

**Supplementary figure S1**. Funnel plot of overall physical activity interventions on self-esteem


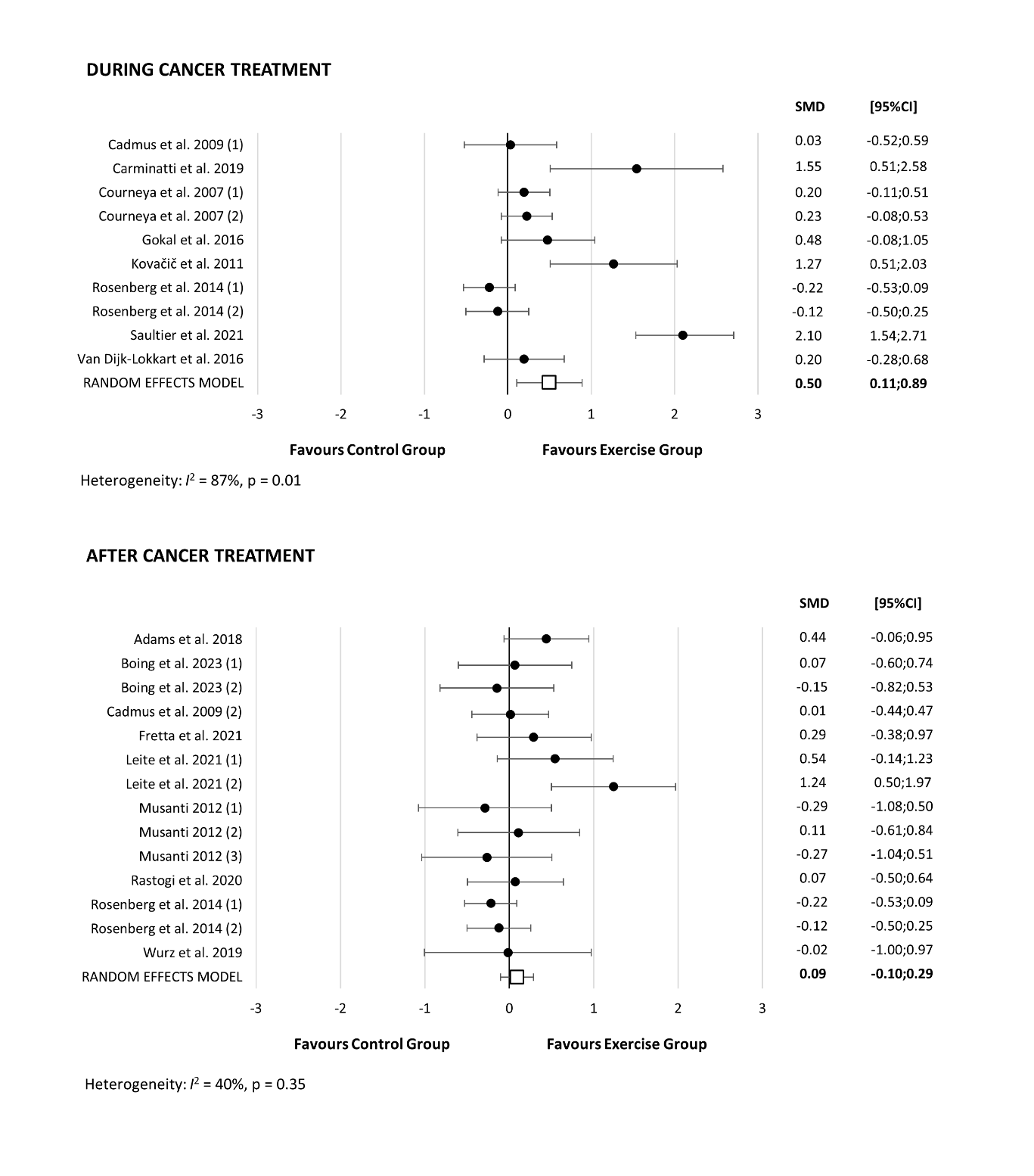
**Supplementary figure S2**. Forest plot of overall physical activity interventions on self-esteem by cancer status

SMD: Standardized mean difference; CI: confidence intervals.

Boing et al. 2023 (1): represents the mind-body exercise; Boing et al. 2023 (2): aerobic physical activity; Cadmus et al. 2009 (1): combined physical activity during cancer treatment; Cadmus et al. 2009 (2): combined physical activity after cancer treatment; Courneya et al. 2007 (1): aerobic physical activity; Courneya et al. 2007 (2): resistance training; Leite et al. 2021 (1): aerobic physical activity; Leite et al. 2021 (2): mind-body exercise; Musanti 2012 (1): aerobic physical activity; Musanti 2012 (2): resistance training; Musanti 2012 (3): combined physical activity; Rosenberg et al. 2014 (1): outdoor adventure 1 (people for whom it was their first outdoor adventure program); Rosenberg et al. 2014 (2): outdoor adventure 2: people for whom it was their second outdoor adventure program.


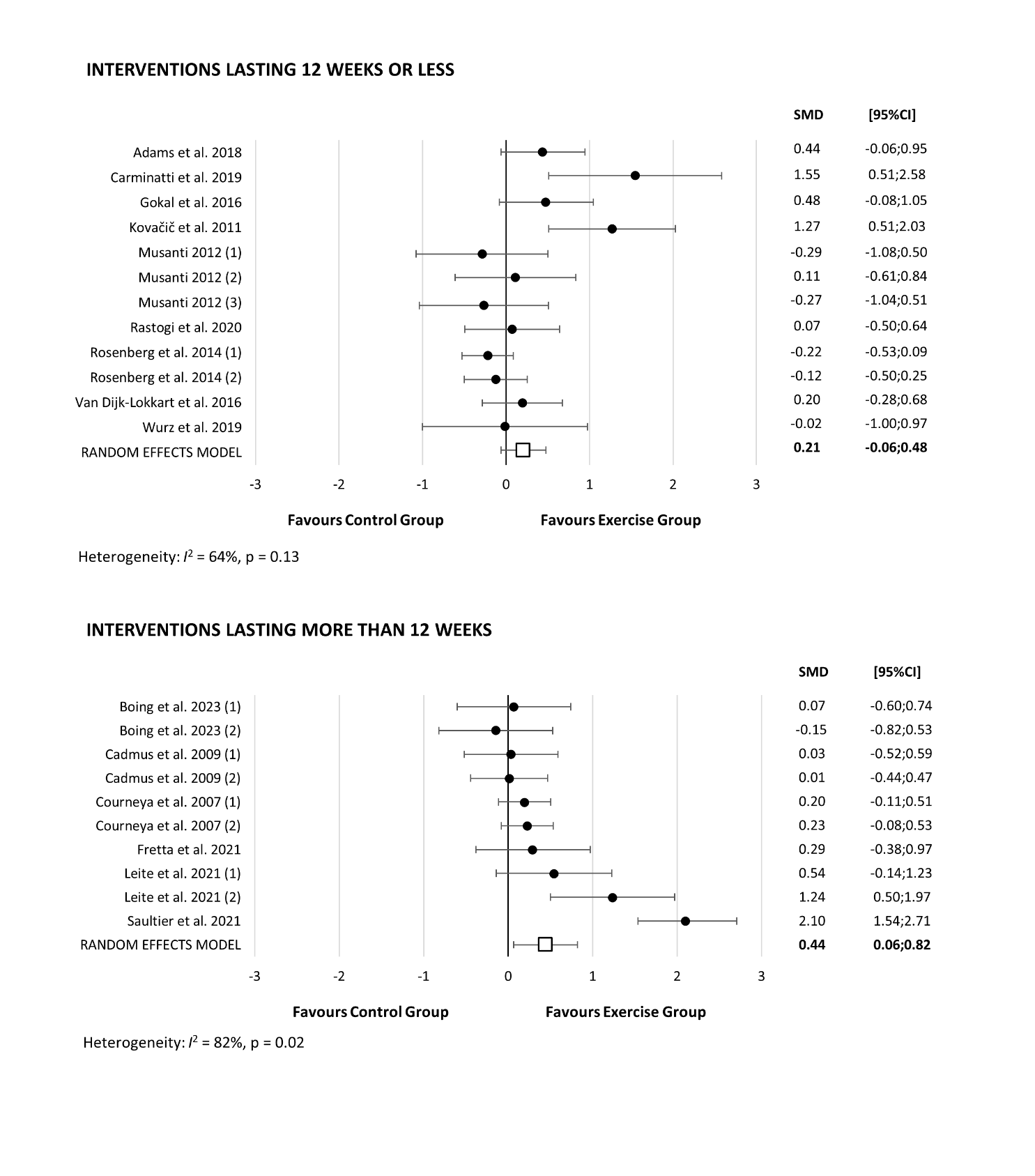


**Supplementary figure S3**. Forest plot of overall physical activity interventions on self-esteem by the length of the intervention

SMD: Standardized mean difference; CI: confidence intervals.

Boing et al. 2023 (1): represents the mind-body exercise; Boing et al. 2023 (2): aerobic physical activity; Cadmus et al. 2009 (1): combined physical activity during cancer treatment; Cadmus et al. 2009 (2): combined physical activity after cancer treatment; Courneya et al. 2007 (1): aerobic physical activity; Courneya et al. 2007 (2): resistance training; Leite et al. 2021 (1): aerobic physical activity; Leite et al. 2021 (2): mind-body exercise; Musanti 2012 (1): aerobic physical activity; Musanti 2012 (2): resistance training; Musanti 2012 (3): combined physical activity; Rosenberg et al. 2014 (1): outdoor adventure 1 (people for whom it was their first outdoor adventure program); Rosenberg et al. 2014 (2): outdoor adventure 2: people for whom it was their second outdoor adventure program.


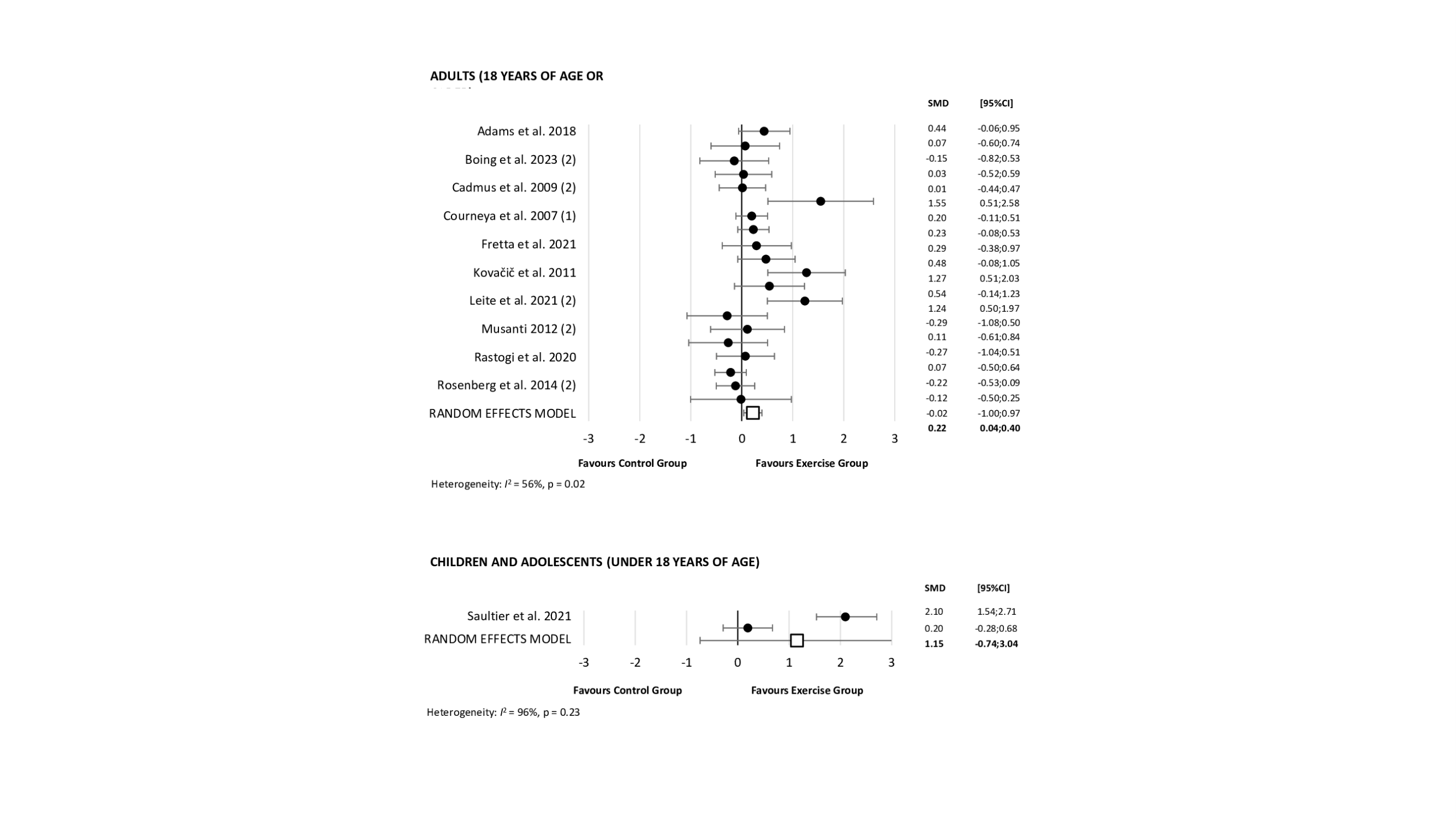


**Supplementary figure S4**. Forest plot of physical activity interventions on self-esteem by age.

SMD: Standardized mean difference; CI: confidence intervals.

Boing et al. 2023 (1): represents the mind-body exercise; Boing et al. 2023 (2): aerobic physical activity; Cadmus et al. 2009 (1): combined physical activity during cancer treatment; Cadmus et al. 2009 (2): combined physical activity after cancer treatment; Courneya et al. 2007 (1): aerobic physical activity; Courneya et al. 2007 (2): resistance training; Leite et al. 2021 (1): aerobic physical activity; Leite et al. 2021 (2): mind-body exercise; Musanti 2012 (1): aerobic physical activity; Musanti 2012 (2): resistance training; Musanti 2012 (3): combined physical activity; Rosenberg et al. 2014 (1): outdoor adventure 1 (people for whom it was their first outdoor adventure program); Rosenberg et al. 2014 (2): outdoor adventure 2: people for whom it was their second outdoor adventure program.


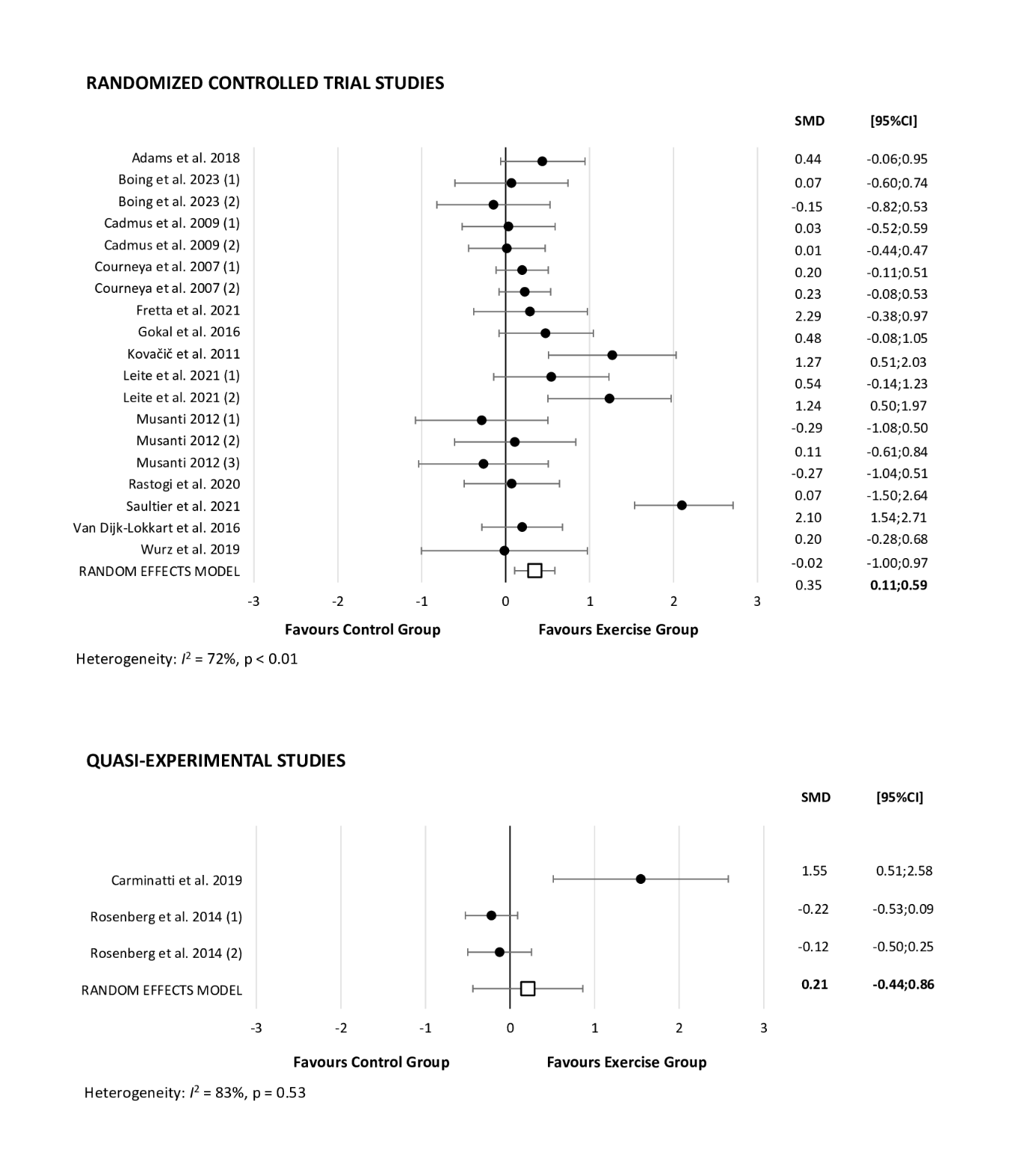


**Supplementary figure S5**. Forest plot of physical activity interventions on self-esteem by type of study.

SMD: Standardized mean difference; CI: confidence intervals.

Boing et al. 2023 (1): represents the mind-body exercise; Boing et al. 2023 (2): aerobic physical activity; Cadmus et al. 2009 (1): combined physical activity during cancer treatment; Cadmus et al. 2009 (2): combined physical activity after cancer treatment; Courneya et al. 2007 (1): aerobic physical activity; Courneya et al. 2007 (2): resistance training; Leite et al. 2021 (1): aerobic physical activity; Leite et al. 2021 (2): mind-body exercise; Musanti 2012 (1): aerobic physical activity; Musanti 2012 (2): resistance training; Musanti 2012 (3): combined physical activity; Rosenberg et al. 2014 (1): outdoor adventure 1 (people for whom it was their first outdoor adventure program); Rosenberg et al. 2014 (2): outdoor adventure 2: people for whom it was their second outdoor adventure program.


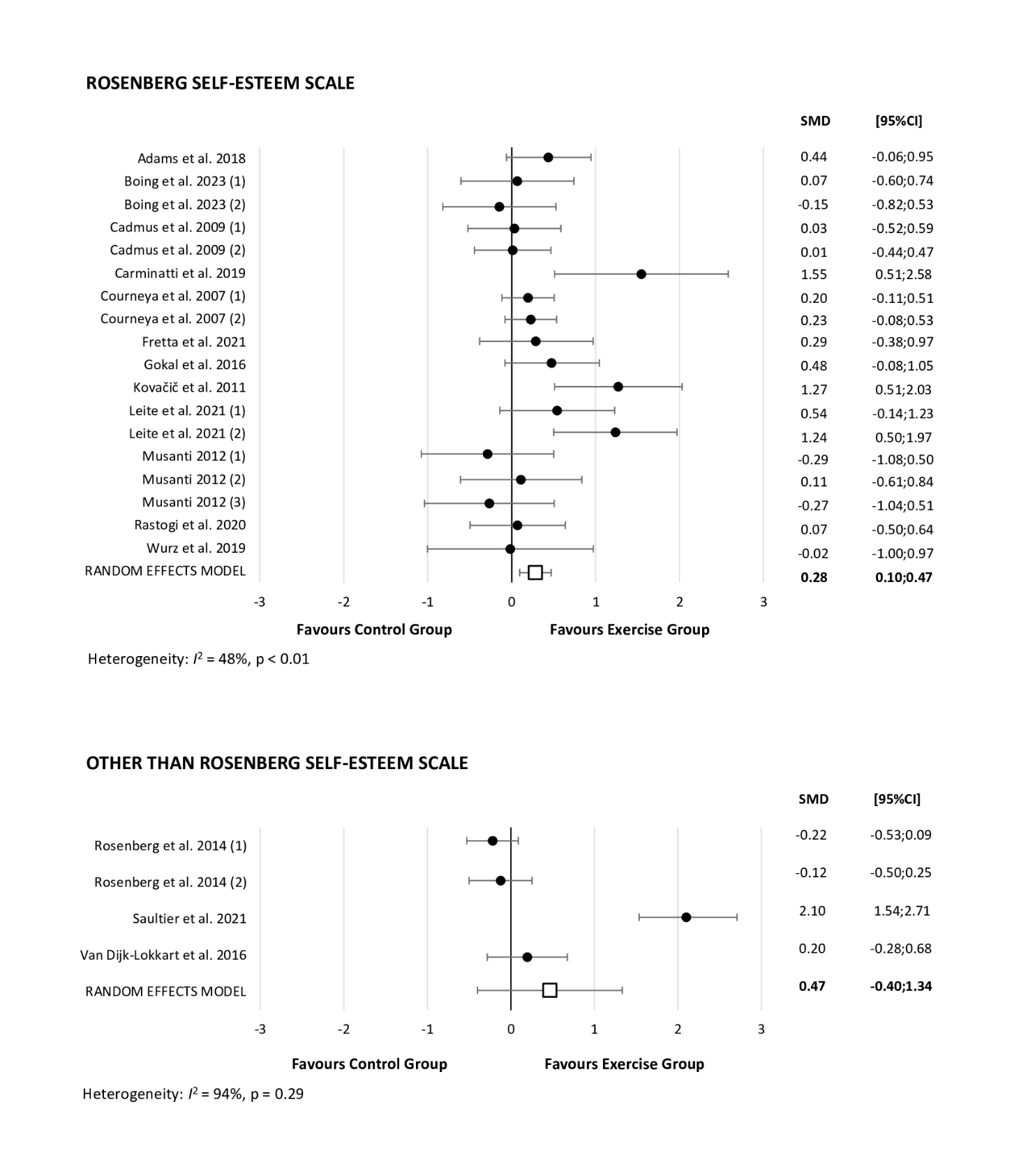


**Supplementary figure S6**. Forest plot of physical activity interventions on self-esteem by type of questionnaire.

SMD: Standardized mean difference; CI: confidence intervals.

Boing et al. 2023 (1): represents the mind-body exercise; Boing et al. 2023 (2): aerobic physical activity; Cadmus et al. 2009 (1): combined physical activity during cancer treatment; Cadmus et al. 2009 (2): combined physical activity after cancer treatment; Courneya et al. 2007 (1): aerobic physical activity; Courneya et al. 2007 (2): resistance training; Leite et al. 2021 (1): aerobic physical activity; Leite et al. 2021 (2): mind-body exercise; Musanti 2012 (1): aerobic physical activity; Musanti 2012 (2): resistance training; Musanti 2012 (3): combined physical activity; Rosenberg et al. 2014 (1): outdoor adventure 1 (people for whom it was their first outdoor adventure program); Rosenberg et al. 2014 (2): outdoor adventure 2: people for whom it was their second outdoor adventure program.
